# Combined application of nasogastric tubes and nasointestinal tubes in neurosurgical intensive care patients with stress ulceration: a novel solution to treatment and early enteral nutrition

**DOI:** 10.1186/s40064-016-3431-7

**Published:** 2016-10-12

**Authors:** Tianshu Lu, Jingyu Guan

**Affiliations:** Department of Neurosurgery, The General Hospital of Shenyang Military Region, No. 83 Cultural Road, Shenyang, 110840 China

**Keywords:** Neurosurgery, Stress ulceration, Nasogastric tube, Nasointestinal tube

## Abstract

**Objective:**

Stress ulcers occur frequently in neurosurgical intensive care patients and can pose serious risks. We summarized the clinical experience of the combined application of nasointestinal tubes for early restoration of enteral nutrition and of nasogastric (NG) tubes for stress ulceration treatment in patients hospitalized in a neurosurgical intensive care unit.

**Methods:**

From January 2011 to June 2011, a series of 67 patients with stress ulceration hospitalized in a neurosurgical intensive care unit were randomized to two groups. The control group (33 patients) received treatment with NG tube decompression and drainage according to international guidelines, and parenteral nutrition was changed to enteral feeding until there was no grossly visible blood in the NG tube. The nasointestinal tube group (34 patients) received treatment combining application of NG tubes and nasointestinal tubes. Drainage was performed with NG tubes as in the control group, with concurrent placement of nasointestinal tubes. Duration until resolution of stress ulceration and days until start of enteral nutrition were compared between the two groups.

**Results:**

Duration until resolution of stress ulceration was 4.5 days in the control group and 4.3 days in the nasointestinal tube group. There was no difference between the two groups (*P* > 0.05). Duration until start of enteral nutrition was 4.5 days in the control group and 1 day in the nasointestinal tube group. There was an obvious difference between the two groups (*P* < 0.01).

**Conclusions:**

The combined application of NG tubes and nasointestinal tubes in neurosurgical intensive care patients with stress ulceration is an effective means of treating stress ulceration and restoring early enteral nutrition.

## Background

Stress ulcer occurs frequently in neurosurgical intensive care patients and can pose serious risks (Liu et al. 2015). Traditionally, stress ulcer in neurosurgical intensive care patients is treated with nasogastric (NG) tube decompression and drainage, in addition to other conventional therapies. However, stress ulcer management via NG tube makes it impossible to administer enteral nutrition via NG tube, and patients must receive parenteral nutrition (Madsen et al. 2014).

Enteral nutrition can provide patients with energy sources and mechanical stimulation to the gastrointestinal tract to prevent intestinal mucosal atrophy, and prevent translocation of intestinal bacteria and endotoxin as a result of intestinal barrier damage (Krag et al. 2013). In addition, enteral nutrition is more economical than parenteral nutrition. The majority of neurosurgical intensive care patients have no intestinal dysfunction, and enteral nutrition is preferable.

The combined application of NG tubes and nasointestinal tubes offers a novel solution to the clinical need for simultaneous stress ulcer management and early restoration of enteral nutrition in patients with brain injury and stress ulcer.

From January 2011 to June 2011, a series of 67 patients with stress ulceration hospitalized in a neurosurgical intensive care unit were randomized to two groups. The control group (33 patients) received treatment with NG tube decompression and drainage according to international guidelines, and parenteral nutrition was changed to enteral feeding until stress ulceration resolution. The nasointestinal tube group (34 patients) received treatment combining application of NG tubes and nasointestinal tubes. NG tube drainage was started as in the control group, with concurrent placement of a nasointestinal tube. Early enteral nutrition via nasointestinal tube was initiated after validating successful nasointestinal tube placement with abdomen and chest X-ray examination performed the second day after placement. Duration until resolution of stress ulceration and days until start of enteral nutrition were compared between the two groups. The clinical experience drawn from the combined application of NG tubes and nasointestinal tubes is summarized.

## Methods

### Patients

From January 2011 to June 2011, a series of 67 patients who were hospitalized in the neurosurgical intensive care unit of our hospital developed stress ulceration. Their Glasgow scores ranged from 4 to 8. The patient population consisted of 37 men and 30 women, aged between 18 and 75 years, including 19 patients with hypertensive basal ganglia hemorrhage, 9 with brain stem hemorrhage, 18 with subarachnoid hemorrhage, 2 with diffuse axonal injury, 15 with cerebral contusion, and 4 with history of intracranial tumor surgery. This study was conducted in accordance with the declaration of Helsinki. This study was conducted with approval from the Ethics Committee of the General Hospital of Shenyang Military Region, Written informed consent was obtained from all participants.

### Grouping and treatment

Patients with stress ulceration hospitalized in the neurosurgical intensive care unit were randomized to two groups. The control group (33 patients) received treatment with NG tube decompression and drainage according to international guidelines, and parenteral nutrition was changed to enteral feeding until stress ulceration resolution. The nasointestinal tube group (34 patients) received treatment combining application of NG tubes and nasointestinal tubes. Drainage was performed with NG tubes as in the control group, with concurrent placement of nasointestinal tubes. Early enteral nutrition via nasointestinal tube was initiated after validating successful nasointestinal tube placement with abdomen and chest X-ray examination performed the second day after placement.

**Fig. 1:**
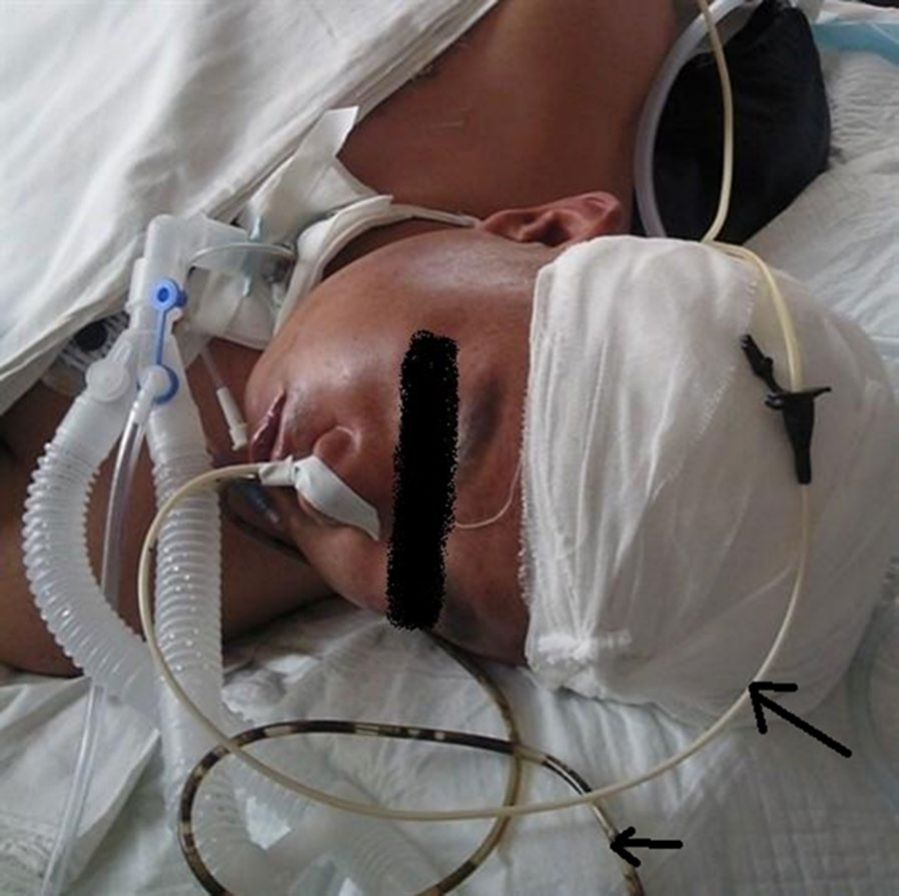
Combined use of nasogastric tube and nasointestinal tube. The *long arrow* shows enteral nutrition through a naso-jejunal tube and the *short arrow* shows nasogastric tube drainage

**Fig. 2:**
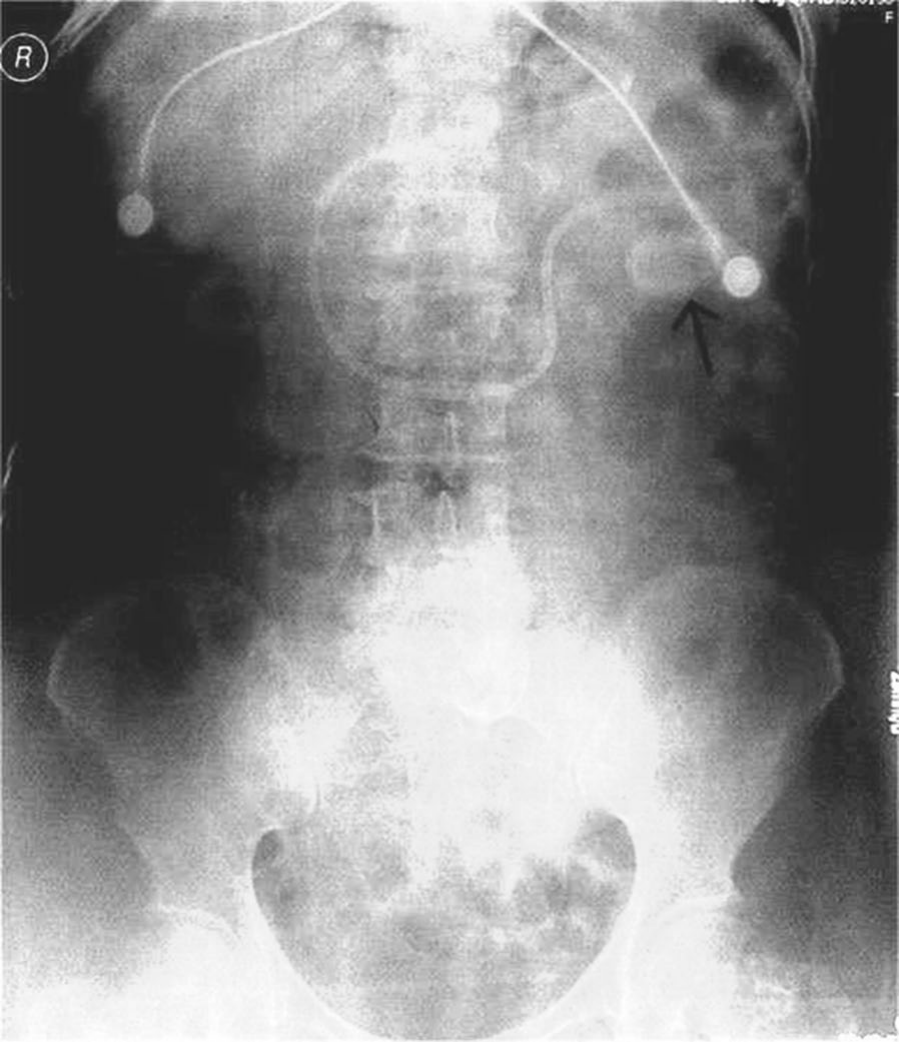
Abdomen and chest X-ray. The *arrow* indicates the tip of the nasointestinal tube within the jejunum

Upon admission, in addition to intravenous administration of hemostatic agents and proton pump inhibitors, the patients who developed stress ulceration were also given cold saline + Yunnan Baiyao via NG tube for an hour 3 times a day, followed by gastrointestinal decompression. Spiral self-propelling nasointestinal tubes were inserted when patients felt no obvious abdominal distension. Intramuscular injection of metoclopramide was performed before placement for safety. Abdomen and chest X-ray examination was conducted the next day to validate that the nasointestinal tubes had been placed within the jejunum and enteral nutrition via the nasointestinal tubes had been established (Figs. 1, 2).

The nutritional treatment regime was as follows. In the nasointestinal tube group, the caloric intake was 20 kcal/kg/days on the day following nasointestinal tube placement; if tolerated, and when patients had stable vital signs, the intake was increased to 30 kcal/kg/days. A peristaltic pump was used for continuous infusion, with the rate initially set at 20 ml/h, which was progressively increased to 50–100 ml/h. Enteral nutrition via the nasointestinal tube was maintained after stress ulceration resolution. In the control group, parenteral nutrition was changed to enteral feeding until stress ulceration resolution.

### Statistical analysis

The data of the two groups were analyzed using SPSS 13.0.

## Results

Duration until resolution of stress ulceration was 4.5 days in the control group and 4.3 days in the nasointestinal tube group. There was no difference between the two groups (*P* > 0.05). The duration until start of enteral nutrition was 4.5 days in the control group and 1 day in the nasointestinal tube group. There was an obvious difference between the two groups (*P* < 0.01).

## Discussion

Stress ulceration has been also referred to as stress gastritis, stress erosive gastritis, and hemorrhagic gastritis (Lucas 1981). Within the first 24 h after intensive care unit (ICU) admission, 75–100 % of critically ill patients have some endoscopic evidence of gastroduodenal or upper gastrointestinal lesions (Shears et al. 2016; Buendgens et al. 2016; Krag et al. 2016). About 50–77 % of critically ill patients with gastrointestinal bleeding will die, typically of the underlying medical condition or of multiple organ failure (Spirt and Stanley 2006). Optimal management of stress ulcer prophylaxis requires a concerted effect among all members of the healthcare team (Marik 2010). In a recent French, multicenter observational study, 32 % of patients hospitalized in the ICU received stress ulcer prophylaxis (Preslaski et al. 2014; Lam et al. 1999; Quenot et al. 2008). Compared with parenteral nutrition, enteral feeding has several advantages for ICU patients, including buffering of acid and acting as a direct source of mucosal energy for the secretion of cytoprotective prostaglandins and mucus, in addition to improving mucosal blood flow (Barletta et al. 2002; Ephgrave et al. 1990; Shorr et al. 1984).

Patients with a functioning gastrointestinal (GI) tract who are malnourished or at risk for the development of stress ulcer are candidates for feeding tube placement. There are several choices of access route and device, which must be tailored to the individual by considering the disease process and how long the patient will probably require nutritional support. NG tubes are used widely and are easily placed, and allow gastric residuals to be checked to assess GI tolerance and pH. Gastric pH monitoring is essential to stress ulcer prophylaxis (Bradley et al. 1998). Some authors believe that the early initiation of enteral nutrition accounts for the low incidence of bleeding from stress ulceration (Faisy et al. 2003). However, the traditional 16- or 18-F NG tube (intended for gastric drainage) is uncomfortable and may promote relatively greater gastroesophageal reflux by holding the lower esophageal sphincter open more than occurs with a narrower tube. Smaller-caliber nasointestinal feeding tubes (e.g., the Dobhoff tube, 8–10 French) are more comfortable and less erosive to the nasopharynx and esophagus, but they can clog when not carefully maintained, and also collapse easily. Generally, there are two methods of intestinal tube placement: one is under gastroscopy, and the other with the aid of contrast agents under X-ray, which requires transporting patients to a radiology department. This is time-consuming and contraindicated for critically ill patients (Heyland et al. 2004). A spiral self-propelling nasointestinal tube can be managed at the bedside, and is a simple alternative for tube placement with high success rates (Wan et al. 2015; Chen et al. 2009). For patients with traumatic brain injury associated with skull base fracture, nasointestinal tubes can be placed together with NG tubes via the patient’s mouth. The combined procedure offers a neat solution for simultaneous pH monitoring and enteral nutrition feeding. Since pulmonary infection is a major complication in seriously ill neurosurgical patients and gastric residual volume is an important factor affecting pulmonary infection, we continue to apply enteral feeding via nasointestinal tubes after ulcer healing. Nasointestinal-tube enteral feeding can effectively reduce gastric residual volume and decrease the incidence of pulmonary infection (Lu 2009). In our study, stress ulcer bleeding in all 30 patients resolved with this treatment in an average of 4.5 days, with no gross blood observed in the NG tube. Meanwhile, these patients were totally dependent on parenteral nutrition for nutritional needs. However, treatment of stress ulcer via NG tubes precludes the administration of enteral nutrition via NG tube, and patients must receive parenteral nutrition.

To our knowledge, this is the first report of the combined application of NG tubes and nasointestinal tubes in neurosurgical intensive care patients with stress ulceration. This is a novel method that can resolve stress ulcers and simultaneously provide enteral nutritional support. This approach may have a number of potential applications for use in severely ill neurosurgical intensive care patients.
